# Green catalyst for clean fuel production via hydrodeoxygenation

**DOI:** 10.55730/1300-0527.3589

**Published:** 2023-10-11

**Authors:** Selva BİLGE, Yusuf Osman DONAR, Samed ERGENEKON, Beyza ÖZOYLUMLU, Ali SINAĞ

**Affiliations:** Department of Chemistry, Faculty of Science, Ankara University, Ankara, Turkiye

**Keywords:** Hydrodeoxygenation, biomass, biooil, biofuel, catalysts and carbon materials, green catalyst

## Abstract

The development of new fuel sources to replace nonrenewable fossil fuels has received substantial attention due to the ongoing demand for fossil fuels. Biomass and raw waste materials are crucial sources to produce suitable alternative fuels instead of nonrenewable fuels and offer a greener approach. Therefore, improving the fuel properties of biooils produced from the thermochemical conversion of biomass and raw waste materials is critical as it is used as an alternative to nonrenewable fuel. Developing an economical and eco-friendly method to produce sustainable and renewable oil by improving biooil containing large amounts of phenolic compounds has become imperative. One of the most intriguing and promising technologies for refining biooil to produce renewable fuels of comparable quality to conventional fossil fuels is the hydrodeoxygenation (HDO)-based process for converting biooil to renewable fuels. This method is almost one of the best improving methods described in the literature. At this point, it is of great importance that the HDO process is carried out catalytically. Carbon materials are preferred for both designing catalysts for HDO and supporting metal nanoparticles by providing chemically inert surfaces and tunable functional groups, high surface area and active sites. The HDO of biomass and raw waste materials has significantly advanced thanks to carbon-based catalysts. In this review, the effect of the surface character and catalytic ability of the carbon support, especially prepared by the green synthesis technique, on the HDO reaction during biooil improvement is discussed. Moreover, HDO reaction parameters and recent studies have been investigated in depth. Thus, green carbon catalysts’ role in clean fuel production via the HDO process has been clarified.

## Introduction

Technological advances and increasing consumption activities worldwide lead to significant global energy demand [1]. Fossil fuels such as oil, coal, and natural gas comprise a large part of the current global energy demand. However, fossil fuels cause problems such as nonrenewability, geopolitical uncertainties, and massive greenhouse gas emissions. For this reason, it is of great importance to evaluate alternative renewable energy sources to meet the increasing energy demand and overcome environmental problems. The process of transforming biomass into usable energy sources such as heat, electricity, and liquid fuels is known as biomass energy. Biomass can also be defined as a renewable energy source that can be produced on demand with uninterrupted advantage, considered the most promising alternative to fossil fuels. An estimated 10 billion tons of biomass are produced annually worldwide, producing more energy than 2 billion tons of conventional coal (dry base) [2]. Basically, biomass resources include all aquatic and terrestrial organisms, plants, residues, and waste derived from them, among them, plants are composed of renewable organic materials and are an ideal source for obtaining clean fuels [3]. The most critical components of plant-based biomass resources are cellulose (35–45 wt.%), hemicellulose (25–30 wt.%), and lignin (20–35 wt.%) [3]. It has the weakest component feature when it decomposes due to the branches in the hemicellulose structure. In this respect, cellulose is much more intense than hemicellulose because long polymers with side changes in cellulose increase the strength of the bonds. Lignin is the most substantial component and has a strong resistance to break down compared to cellulose and hemicellulose because of its complicated structure, mostly made up of numerous aromatics with side chains [1]. Techniques such as gasification, hydrolysis, and pyrolysis are used to produce liquid fuels from biomass [1]. It is possible to obtain three different biofuels, first and second generation, from biomass. First-generation biofuels, also known as original biofuels, are produced from staple food crops such as wheat, corn, grain, sunflower, sugarcane, potatoes, and coconuts [1]. Second-generation biofuels are produced from lignocellulosic-based agricultural and forestry raw materials, construction, industrial and municipal waste [1]. Finally, it is known that third-generation biofuels are generally obtained from algae-based biomass [1]. At this point, using green technologies to convert biofuels, lessen their negative effects, and assist with the disposal issue is promising [4]. The pyrolysis technique is a sustainable and relatively more environmentally friendly process performed at temperatures of 300–600 ºC in an oxygen-free atmosphere to thermally decompose biomass into rich hydrocarbon liquid fuel, coal, and syngas [5]. The pyrolysis process is based on the degradation of lignin, cellulose, and hemicellulose in the biomass content into long polymer chains and short carbon-carbon range hydrocarbons. Most existing studies in the literature describe biomass pyrolysis as a complex process due to different feedstock structures and multiple reactions cooccurring in the reactor [5]. Many studies have shown that oxygenated compounds such as guaiacol, phenol, cresol, anisole, eugenol, and acetic acid are found in biomass-based pyrolysis oil. The presence of these compounds increases the oxygen atom content and acidity of the pyrolysis oil and deterioration of its calorific value and viscosity. The removal of these oxygen compounds is important in reducing the corrosiveness of the pyrolysis oil and making it suitable for modern engines. However, despite many studies on improving biooil properties and different methods such as distillation [6], filtration [7], emulsion [8], esterification [9], catalytic pyrolysis [10], zeolite-cracking [11], aldol condensation [10], and ketonization [12], the improvement in the fuel properties of biooil still has not reached the desired targets. Recently, studies in the field of improving the thermal value and thus its potential use as a fuel by removing the high oxygen content of biooil with the HDO method have increased significantly. HDO; in the presence of a catalyst, in a hydrogen atmosphere, and partially at elevated temperatures (200–400 °C), including reactions such as deoxygenation (C-O bond breaking), hydrogenation (hydrogenation of the C = O bond and aromatic ring), hydrogenolysis, hydrocracking, decarboxylation and dehydration, and thus, it is a critical method that makes it possible to obtain hydrocarbons from oxygenated compounds [13]. The type of pyrolysis oil produced determines the optimal HDO operation conditions. To improve the biooil effectively, temperature, pressure, and catalyst type are known as the main variables for optimization [14]. The catalyst type plays a critical role in increasing the biooil yield and oxygen removal and increasing the hydrocarbon content. In some studies, it has been reported that carbon-based heterogeneous catalysts are critical in terms of selective and rapid conversion of lignin to valuable chemicals [3]. When used as a catalyst support material, they also offer the benefit of recycling the catalyst without harming the metal, and these characteristics draw the interest of scientists worldwide. Carbon materials have superior physicochemical properties such as unique electrical conductivity, excellent chemical stability, high specific surface area, tunable surface functional groups, and multiple pore structures [3]. Moreover, their high thermal stability, three-dimensional structure, high reusability, and interconnected pores make them easy to act as catalyst support [3]. According to their crystal structures, they have different allotropic forms such as carbon black, activated carbon, graphite, diamond, carbon aerogel, carbon nanofibers, carbon nanotubes, fullerene, graphene and its derivatives [3]. Among these carbon materials, activated carbons called biochar, prepared with relatively greener synthesis techniques such as hydrothermal carbonization and pyrolysis, offer a much green approach for the HDO processing of biooil.

**Figure 1:**
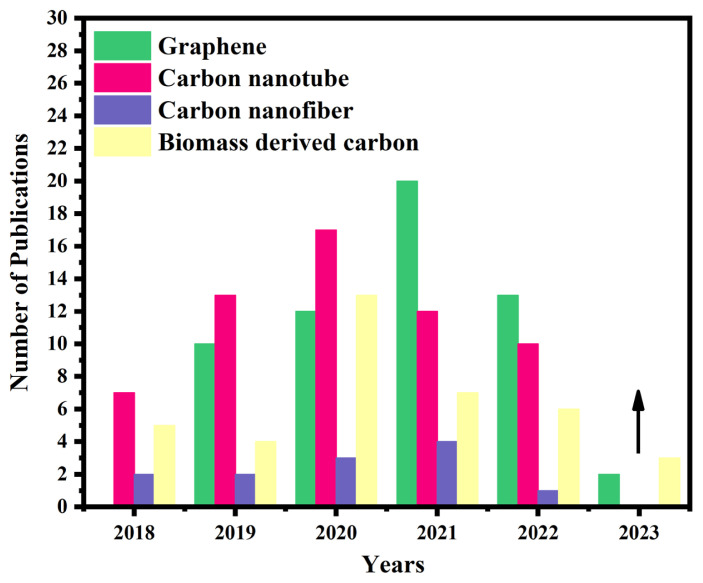
The role of different types of carbon in HDO applications for catalysts (collected from ISI Web of Knowledge, March 2023).

Figure 1 shows the studies that have been carbons such as graphene, carbon nanotube, carbon nanofibers, and biomass-derived carbon as catalysts in HDO applications in the last five years in the literature. Literature reviews indicate that commercial carbon materials are used in HDO applications but are also increasing in research for biomass-derived carbon. When Figure 1 is examined, it is seen that the interest in the use of biomass-derived carbon as a catalyst increased, especially in 2020.

The results show that catalytic-based green HDO studies are among today’s hot topics, as they reveal the potential use of biooils as a clean fuel.

## 2. A brief overview of HDO

HDO is a chemical reaction that removes oxygen atoms from organic molecules using hydrogen gas under high temperatures and pressure [15]. This process is commonly used to convert biomass, such as plant or animal fats, into valuable products such as biofuels or chemicals [15]. HDO is considered a promising technology in the field of sustainable energy and is being actively researched to reduce our dependence on fossil fuels [15]. The basic steps of the HDO process are as follows:

- **Bioraw material preparation:** The bioraw material should be appropriately collected, prepared, and treated to remove impurities and moisture and converted into smaller molecules by processes such as pyrolysis [16,17].
- **Catalyst selection:** The choice of catalyst is critical in HDO because it determines the reaction rate, selectivity, and efficiency. Common catalysts used in HDO are metal sulphides and carbides [17].

The choice of catalysts for HDO depends on the activity, stability, selectivity, high surface area, and porosity size of the metal and support catalyst [18]. Common catalysts used in HDO are metal sulphides and carbides [17].

As support material, oxides (Al_2_O_3_, TiO_3_, ZrO_2_, SiO_2_), mesoporous materials (HMS, SBA-15, KIT-6, MCM-41), mixed oxides (Al_2_O_3_-TiO_2_, TiO_2_-SiO_2_, TiO_2_-ZrO_2_, SiO_2_-Al_2_O_3_, ZrO_2_-SiO_2_), active carbon, composites (Al_2_O_3_.MCM-41), zeolites (NaY, USY, Beta, ZSM-5) can be used. A catalyst can be developed using active metal (nickel, cobalt, molybdenum, tungsten, iron, noble metals) that will then be dispersed on these support materials [17].

**Figure 2:**
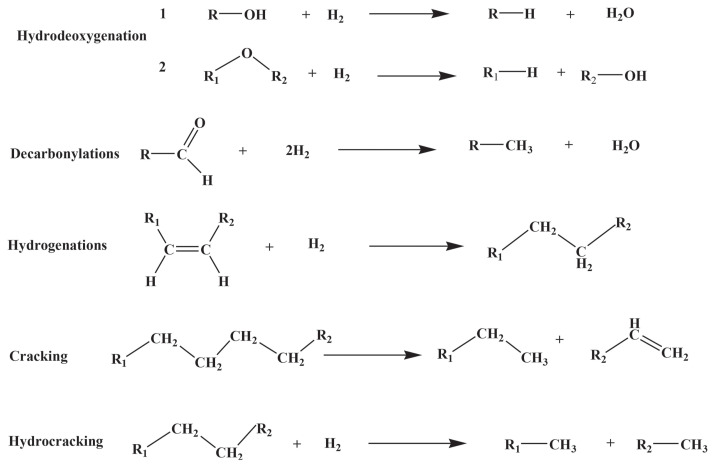
Reactions occurring during the HDO process. Adapted from Ref. [19] with permission from Elsevier.

During the HDO process, decarbonylation, hydrogenation, cracking, hydrocracking, and dehydration reactions occur [19]. In the HDO process, catalysts enhance hydrogenation and deoxygenation reactions, which indicates that the reactions occur on the active metallic sites, with acidic sites [20]. Therefore, HDO method is more advantageous and comprehensive than other methods [13]. The reactions occurring during HDO are given in Figure 2.

In the literature, HDO of substances such as guaiacol, vanillin, furfural is frequently encountered [21–23]. For example, Lan et al. in 2018 investigated the deactivation mechanism of Ni_2_P/SiO_2_ catalyst in guaiacol HDO. In this study, it was revealed that guaiacol is converted to phenol and anisole by demethylation and dihydroxylation, and then to benzene by demethylation of anisole or dihydroxylation of phenol [24].

Similarly, in the study of Lin et al. in 2011, HDO of guaiacol was investigated using two different catalysts, namely Rh-based and sulphided CoMo and NiMo. It has been reported why monometallic and bimetallic Rh catalysts show higher activity than CoMo and NiMo catalysts. The highest guaiacol cyclohexane conversion was obtained in the Rh/ZnO_2_ noble metal catalyst. It has been emphasized that the HDO mechanism of guaiacol consists of two steps: hydrogenation of the guaiacol benzene ring, followed by deoxylation and dehyroxylation of oxygenates. For sulphated CoMo and NiMo/Al_2_O_3_, HDO treatment started with demethylation, demethylation, and deoxygenation, followed by benzene ring formation. In line with these data, it has been reported that the Rh-based catalyst exhibits the best HDO activity. It was emphasized that the classical sulphated CoMo and NiMo catalysts showed the potential to form aromatic structures [25].

In the study of Lan et al. in 2021, the effect of furfural HDO-metalphosphorus stoichiometry on the catalytic properties of silica supported metal phosphites was investigated. When a series of transition metal phosphites were evaluated in the furfural HDO reaction, the HDO activity of furfural was determined as Ni_2_P = MoP > Co_2_P > WP = CuP > Fe_2_P. Furan ring hydrogenation and ring opening are inhibited by increasing P/metal content. It was emphasized in the study that carbonyl conversion increased with higher P/metal ratio [26].

In similar fashion Xu et al. reported that guaiacol converted to cyclohexanol through two parallel ways: demethoxylation followed with hydrogenation (path 1) and the saturation of benzene ring followed with the demethoxylation (path 2) [27].

- **Selectivity and activity:**
- HDO of guaiacol was carried out by Zhou et al. in 2017 on γ-Al_2_O_3_ and ZSM-5 supported catalysts with Ni and Co as active metals. Among these catalysts, NiCo/γ-Al_2_O_3_ catalysts exhibited up to 96.1% better conversion of guaiacol to cyclohexanol as the main product. It can be concluded that the support material has a great influence on both guaiacol conversion and product selectivity. Synergistic effects between Ni and/or Co and support (γ-Al_2_O_3_ and ZSM-5) were investigated for catalytic activity, indicating that appropriate acidity and interaction between metal particles and support are beneficial for HDO of guaiacol. NiCo/γ-Al_2_O_3_ catalysts have been reported to exhibit better catalytic activity for the HDO of guaiacol, due to the increased catalytic activity as well as the incorporation of Co into Ni/γ-Al_2_O_3_, increasing the acidity, reducibility, and metal particle dispersion of the catalyst [28].
- **Reaction conditions:** The reaction is typically conducted at high temperatures and pressure in the presence of hydrogen gas to promote deoxygenation [29]. The reaction time can vary and be optimized based on the raw material and catalyst used.
- **Product separation and refining:** Product separation and refining are necessary to isolate and purify the desired target molecules [30]. The break can be based on the physical and chemical properties of the products, including boiling point, solubility, and polarity [31].
- **Byproduct utilization:** As with any chemical reaction, HDO often yields byproducts that can be used in coproducts applications, including fuel, feedstock for downstream processes, and value-added chemicals.
- Overall, the HDO process requires carefully selecting raw materials, catalysts, and reaction parameters to achieve maximum efficiency and product output. The reaction parameters of HDO can vary depending on the specific material being converted and the desired product. However, some general parameters may include:
- **Temperature:** HDO typically occurs at high temperatures ranging from 200–400 °C [32].
- **Pressure:** High-pressure conditions, typically ranging from 30–150 bar, are required to favour complete conversion of the reactants [33].
- **Hydrogen pressure:** The hydrogen pressure plays a crucial role in determining the conversion efficiency and selectivity of the HDO reaction.
- **Catalyst properties:** The choice of catalyst and its properties such as surface area, acidity, and metal content can affect the HDO reaction [34].
- **Solvent system:** Depending on the reactant and catalyst properties, specific solvents may need to be used to facilitate HDO reactions [35].

These parameters can be altered and optimized based on the requirements for specific HDO reactions, depending on the bioraw materials and target products. Therefore, it is crucial to examine these parameters by comparing them with the current literature.

## 3. Advantages and challenges of HDO processing

HDO is an effective method for reducing the oxygen content of organic molecules [36]. This is important because oxygen can decrease fuel energy density and reduce chemicals’ stability and shelf-life [36]. Also, this method can convert oxygen-containing molecules into hydrocarbons with a higher energy density, making them better suited for transportation fuels [36]. Clean fuels produced through HDO have lower sulphur and nitrogen content levels than crude oil, reducing emissions of harmful pollutants when burned [36]. Thus, HDO helps reduce the dependency on fossil fuels by producing renewable and clean fuels from biomass [36]. On the other hand, this method can use a wide variety of biomass-derived feedstocks, including agricultural and forestry residues, energy crops, and municipal waste. Overall, the HDO process can offer significant advantages to produce cleaner, more efficient fuels and chemicals [37]. Still, it also has some critical drawbacks that need to be carefully considered before it can be widely adopted. For instance, one of the most significant drawbacks is catalyst poisoning [38]. Catalysts used in HDO can be easily poisoned by contaminants in the feedstock, reducing their effectiveness and lifespan [38]. Additionally, considering the environmental impact, the production of hydrogen gas, which is an essential component of the HDO method, can lead to the formation of greenhouse gases and other harmful emissions [37].

## 4. HDO of biomass-derived oxygenates on heterogeneous catalysts

The mechanism of HDO conducts the adsorption of the oxygenate molecule onto the catalyst surface, followed by hydrogenation of the oxygen-containing functional group (such as -OH, -COOH, or - C = O) to form a hydroxyl group (-OH) or a carbonyl group (-C = O). This intermediate is then further hydrogenated to remove the oxygen atom and form a hydrocarbon. Heterogeneous catalysts play a crucial role in HDO reactions, as they facilitate the removal of oxygen atoms from the oxygenates and promote the formation of C-C bonds to produce hydrocarbons [39]. The catalysts used in HDO reactions are typically composed of metals such as nickel, cobalt, or palladium supported on various materials such as alumina, silica, or zeolites. The selection of catalyst and reaction conditions is related to the specific oxygenate used as the feedstock. For example, lignin-derived phenolic compounds are typically more challenging to convert to HDO than carbohydrate-derived oxygenates, requiring higher temperatures and pressures, longer reaction times, and more active catalysts. Generally, the HDO reaction can be divided into two main steps: [1] activation of the oxygenate molecule by the catalyst surface, and [2] hydrogenation and deoxygenation of the activated species to produce the desired hydrocarbon product [40]. The mechanism of these steps depends on the specific catalyst and reaction conditions and can involve various intermediates and reaction pathways. In conclusion, HDO on heterogeneous catalysts has shown great potential for producing renewable biofuels and chemicals from biomass-derived oxygenates [39]. However, there are still many challenges to overcome, such as improving the selectivity and activity of the catalysts, reducing the cost of the process, and developing more efficient and sustainable sources of hydrogen gas.

### 4.1 Other metal catalysts

Several different metal catalysts have been investigated and utilized in HDO processes. The parameters of the reaction, the composition of the feedstock, the desired product, and the process’s needs all influence the catalyst’s choice. Catalysts play an important role in the HDO reaction. Different catalysts lead to the formation of different products, allowing the discovery of superior catalytic properties and HDO mechanisms. Therefore, it is important to understand the mechanism of the catalytic HDO reaction and the influence of different factors on catalytic activity and product selectivity. Experimental design and catalyst synthesis play an important role and lead the way in improving HDO yield. Some important metal catalysts are frequently used in the literature. In HDO processes, noble metals such as platinum (Pt), palladium (Pd), and ruthenium (Ru) have demonstrated strong catalytic activity. The order of catalytic activities of these metals is Pd > Pt > Rh > Ru [41]. In the HDO reaction, the Pd catalyst is advantageous for decarboxylation, while the Pt catalyst is more advantageous than others for decarbonylation. This function is attributed to the dissimilar adsorption capacities of Pd and Pt catalysts for the hydrolysate fatty acids, as well as the distinct intermediate formation. The d electron orbit of precious metals is not filled, and the reaction species can easily adsorb on the surface. As a result, it exhibits significant catalytic hydrogenation activity at low temperatures. Due to their high cost, they are often used in combination with other metals or as bimetallic catalysts to optimize performance and lower overall catalyst costs. Bimetallic catalysts, composed of two distinct metals, have become popular in HDO applications [42]. Combinations of noble metals (e.g., Pt, Pd) with less expensive transition metals (e.g., Ni, Co) have demonstrated enhanced catalytic properties, including increased selectivity and stability. The combination of different metals is predicted to support catalytic reactions synergistically and improve the performance of the catalyst. However, while noble metals such as Pt and Pd, catalysts for HDO, and their binary combinations are widely used, there are also nonnoble metal catalysts with promising results. These are phosphide, carbide, and nitride, sulphide, oxide, and transition metal catalysts, respectively [1]. For example, specific transition metal nitrides such as molybdenum nitride (MoN) and tungsten nitride (WN) show potential as HDO catalysts. These nitrides have enhanced surface properties and high surface areas that increase their catalytic activity and can effectively support deoxygenation reactions [43]. On the other hand, sulphides such as molybdenum sulphide (MoS_2_) and tungsten sulphide (WS_2_) exhibit superior catalytic activity and selectivity for various biomass conversion reactions [1].

### 4.2 Metal-support interactions for HDO

Strong metal-support interactions have a significant impact on the electronic properties of metal species, thereby influencing their catalytic behaviour [44]. Lots of carbon-based supports show type I isotherms, which indicate microporous structures. During the metal support interaction, metallic particles adsorb into the microporous of the carbon support [44]. The acidity of the carbon support also plays an important role in this interaction [43]. This interaction usually results in a reduction of the surface area of the carbon support material and the overall pore volume. On the other hand, the high dispersion of metal particles on the carbon support material is extremely important for catalytic activity. To achieve acceptable levels of catalyst (support + metal) surface area despite the reduction in surface area after metal-support interaction, the support material must have a high surface area. In some cases, dissolved metal salts are adsorbed onto the support material and then reduced to metal by interaction with the support material [45]. In this case, higher metal dispersion can be achieved. High metal dispersion is extremely important as it provides a higher active metal surface area. Heteroatoms containing carbon supports provide better interactions thanks to the higher electronegativity of heteroatoms such as N, S, or O. These features can be utilized to create metal nanoparticles with strong adhesion and high dispersion. Pd adsorbed on N@CNTs provides electrons to pyridinic N, resulting in electron deficiency in Pd. The N containing carbon nanospheres on a nickel catalyst has a significant influence on reactant adsorption and metal sintering [46]. N-doping in carbon nanospheres reduces the graphitic nature of the catalyst, and quaternary N provides an electron-rich carbon surface, which enhances the metal’s mobility and subsequent sintering [47]. Support surfaces containing heteroatoms can function as defect locations. It improves the catalyst’s hydrophilicity and wettability. The greater the hydrophilicity of the catalyst, the greater its activity for the HDO of vanillin in a polar protic solvent such as water [48]. In a similar manner, microspheres were utilized to support Pd nanocages to modulate its catalytic performance in semihydrogenation of alkynes. At the Pd/N–C interface, the Mott-Schottky effect decreased the electron density of Pd nanocages, which was advantageous for the reaction [49].

## 5. HDO of biomass-derived oxygenates on homogeneous catalysts

In theory, both hetero- and homogeneous catalysts can be used, but since the pioneering work of Schiavo et al. [50] heterogeneous systems have dominated research and development. Although homogeneous catalysts have some advantages over heterogeneous catalysts in terms of catalyst deactivation in the HDO of sugars, sugar alcohols, and their condensates, homogeneous systems also confront unique obstacles in the design of ligands, catalysts, and processes [51]. Since water is an important byproduct of acid-catalysed dehydration steps, the complexes to be used as homogeneous catalysts must be resistant to water and an acidic environment [52]. Complexes to be used as homogeneous catalysts in HDO reactions should be designed to be redox and thermally stable. Otherwise, it can function as a heterogeneous catalyst due to the decomposition of ligands or reduction to bulk metal form. Ligands play a major role at this step [53]. One of the major disadvantages of homogeneous catalysts is the difficulty of recovering and recycling the catalyst, often from very complex reactions and product mixtures. This limits the economically and technically viable application of homogeneous catalysts. Heterogeneous catalysts are more sensitive to poisons, coking, and fouling compared to homogeneous ones. Diffusion limitations between two or more phases can be a limited factor in HDO reactions. On the other hand, the low stability against temperature and aqueous acids of homogeneous catalysts also limits their use [51]. However, effective use of homogeneous catalysts on complex substrates such as real biooil may arguably be even more difficult. Researchers who overcome the difficulties associated with designing homogeneous catalysts for HDO reactions will discover unexplored research area. However, only a small number of researchers have been able to achieve this to date.

## 6. Differences between biomass-based carbon and metal catalysts

Biomass-based carbon catalysts have several advantages over traditional metal-based catalysts. They are abundant, renewable, and inexpensive and can also be produced from the same feedstocks used to create the oxygenates, making them a potentially sustainable and eco-friendly option [54]. The mechanism of HDO on biomass-based carbon catalysts is very similar to that in metal-based catalysts, which involves the adsorption of the oxygenate molecule onto the catalyst surface followed by hydrogenation and removal of the oxygen atom [55]. The active sites for HDO on biomass carbon catalysts are predicted to be surface functional groups such as carboxylic acids, phenolic groups and quinones [56]. Also, the reaction conditions of HDO on biomass-based carbon catalysts are similar to those used for metal-based catalysts using hydrogen gas as the reducing agent. However, biomass-based carbon catalysts may require higher temperatures and longer reaction time than metal-based catalysts due to their lower activity. Another important point is that the HDO process can be selectively carried out. The selectivity of HDO over biomass-based carbon catalysts can be easily controlled by adjusting the biomass feedstock and reaction conditions used to produce the catalyst. For instance, catalysts made from lignin-rich feedstocks may have higher selectivity for aromatic hydrocarbons, while those made from cellulose-rich feedstocks may have higher selectivity for alkanes [57–59]. In this context, HDO on biomass-based carbon catalysts is a promising technology for converting biomass-derived oxygenates into value-added hydrocarbon fuels and chemicals [60]. However, more research is needed to optimize the catalyst design and reaction conditions for specific feedstocks and product applications.

## 7. Biomass carbonization with green synthesis techniques

Biomass carbonization by green synthesis techniques typically involves using renewable and sustainable resources to convert biomass into carbon materials without harmful chemicals or high-energy inputs. Green synthesis techniques used for biomass carbonization include hydrothermal carbonization (HTC), pyrolysis, solvothermal synthesis (SS), and microwave-assisted carbonization (MAC). The HTC technique is based on the principle that biomass reacts with water under high temperatures and pressure to produce carbon materials [61–63]. The process is environmentally friendly as it requires no chemical catalysts or additional energy input. The solid carbonaceous material produced by HTC is highly stable and can be used for long-term carbon sequestration. This can help mitigate greenhouse gas emissions and contribute to climate change mitigation. On the other hand, the pyrolysis technique involves the thermal decomposition of biomass in an oxygen-free environment to produce carbon materials [64–66]. Green pyrolysis techniques use low temperature or slow pyrolysis, which requires lower energy inputs and have fewer emissions than conventional pyrolysis technique [67]. Solvothermal synthesis, another important synthesis technique, is based on the reaction of biomass with a solvent under high temperature and pressure to produce carbon materials [68]. This eco-friendly technique uses biodegradable solvents and requires no additional energy input. Another synthesis technique that has attracted the attention of researchers recently is the microwave-assisted carbonization technique. This carbonization technique involves using microwave to heat and carbonize biomass [69]. The technique can be considered as green because it requires less energy and produces fewer emissions than traditional carbonization methods. Ultimately, green synthesis techniques for biomass carbonization offer a sustainable and environmentally friendly alternative to traditional carbonization methods. These techniques can produce carbon materials with unique properties and various applications in energy storage, catalysis, and environmental remediation.

## 8. Effect of biomass-based carbon materials on the HDO reaction mechanisms

Features of biomass-based carbon materials can significantly impact the HDO reaction mechanisms, and understanding these effects is essential for developing efficient and selective HDO catalysts. Biomass-based carbon materials such as activated carbon can act as catalyst support due to their high surface area, porosity, and chemical properties. Moreover, these materials can provide suitable sites for the adsorption and activation of reactants and intermediates in the HDO reaction, while enhancing species’ mass transfer and distribution. Additionally, studies have demonstrated that the HDO reaction mechanism is directly affected by the type and properties of the biomass-based carbon materials used. For example, carbon materials with high surface acidity and basicity are thought to increase the HDO reaction rate as they can activate reactants and form desired products. On the other hand, carbon materials with low surface reactivity can result in lower HDO yields. Generally, the effects of biomass-based carbon materials on the HDO reaction mechanisms can be summarized as follows:

**Surface area:** Biomass-based carbon materials typically have a high surface area, which can increase the number of active sites available for the HDO reaction [70]. This can lead to higher conversion rates and improved selectivity toward desired products.

**Porosity:** The porosity of biomass-based carbon materials can affect the diffusion of reactants and products, which can impact the reaction kinetics and selectivity [3]. For example, highly porous carbon materials can provide more surface area for the adsorption of reactants, leading to faster reaction rates.

**Functional groups:** Biomass-based carbon materials can contain various functional groups, such as hydroxyl (-OH) and carboxyl (-COOH) groups, which can act as catalytic sites for the HDO reaction. These functional groups can promote the dissociation of oxygen-containing compounds and facilitate the removal of oxygen [71].

**Oxygen content:** The oxygen content of biomass-based carbon materials can also influence the HDO reaction mechanisms. Carbon materials with high oxygen content can act as oxygen donors and facilitate the formation of hydrogen bonds between reactants and products, which can affect the selectivity toward desired products [72].

In summary, using biomass-based carbon materials in HDO reactions can significantly affect the reaction mechanism and overall process efficiency. Therefore, the physicochemical properties and characteristics of these carbon materials should be carefully considered in the design and reaction conditions of HDO catalysts.

## 9. Effect of biomass-based carbon materials on the HDO reaction mechanisms

The microstructure of carbon materials, such as their pore size distribution, surface area, and conductivity, plays a vital role in optimizing the properties of carbon-based catalysts. High dispersion of metal-active species requires a large surface area, which is only possible with the presence of micro- and mesopores. The proper distribution and dispersion of metal active phases on carbon supports increases their accessibility to reactants and decreases diffusion limitations [2]. The introduction of heteroatoms into carbon surfaces adjusts hydrophilicity, acidity, and alkalinity, thereby enhancing the dispersion of metal active phases and the interaction with reactants, especially in polar solvents [73]. In addition, the presence of oxygen-containing functional groups promotes dehydration and the activation of reactants via a synergistic effect with metal active phases and serve as active sites for catalytic reactions [74]. Coal is one of the unique natural substances with a three-dimensional (3D) interconnected network [75]. The organic components of coal are derived from plant remains that underwent decomposition and complex physical and chemical transformations after being buried in the earth. From this point of view, the process of obtaining carbon material from biomass can be considered as the previous step of the process of obtaining carbon material from coal. Coal is already a part of the transition from biomass to carbon material done by nature. Carbonization processes carried out under high pressure and high temperature simultaneously, such as hydrothermal carbonization, are a mimetic of the carbonization process in nature. Various carbon materials, including porous carbon, fullerene, carbon nanotubes, carbon spheres, carbon fibres, graphene, and carbon dots, can be produced using coal, depending on the applied technique. Shale oil can also be used to obtain carbonaceous materials. Compared with conventional crude oil, shale oil contains fewer light components and has higher heteroatom content, which make its processing difficult [76]. On the other hand, high heteroatom content makes oil-shale based carbonaceous materials favourable. The carbonization technique used in the synthesis of advanced carbon-based materials is far more essential than the choice of starting material (coal, oil, or biomass). Using the appropriate techniques, it is possible to obtain carbon material with a high surface area, heteroatom content, surface functional groups, and aromatic skeleton structure from all the mentioned carbon sources. For instance, surface functional groups are observed in carbon-based materials produced by hydrothermal carbonization; however, additional surface functionalization stages are required for high temperature processes such as pyrolysis. Conversely, while a graphitized carbon-based material with a high surface area can be obtained via an ionothermal carbonization process, additional surface activation or graphitization stages may be required in processes such as HTC and pyrolysis.

## 10. Recent applications of HDO for clean fuel

HDO studies carried out using biomass-based carbon materials in the last five years, and the experimental parameters of these studies are summarized in Table. When the studies are examined, notably plant-based wastes are used as a biomass source for HDO catalysts. Additionally, the studies mostly carbonized biomass resources by HTC and pyrolysis techniques. These two techniques are more preferred because they both have high carbon yield, that is, the ability to produce a large amount of carbonized material from the original biomass feedstock. This is important because higher carbon yield means that less raw material is needed to make the same carbonized material. Moreover, when Table is examined, it is observed that mostly vanillin and guaiacol are used as HDO reactants. Vanillin and guaiacol are simple model compounds widely used in HDO studies because they are easy to handle, and their structures represent the oxygen-containing functional groups in lignin. This allows researchers to study the HDO process in a simplified system and better understand the reaction mechanism and kinetics involved in the process. Additionally, vanillin and guaiacol are commercially available and relatively inexpensive, which makes them convenient model compounds for HDO studies. They also react differently toward HDO, with guaiacol being more reactive due to the presence of the methoxy group, which is easier to remove than the hydroxyl group in vanillin. This makes them helpful in comparing the performance of different catalysts and reaction conditions for HDO.

**Figure 3:**
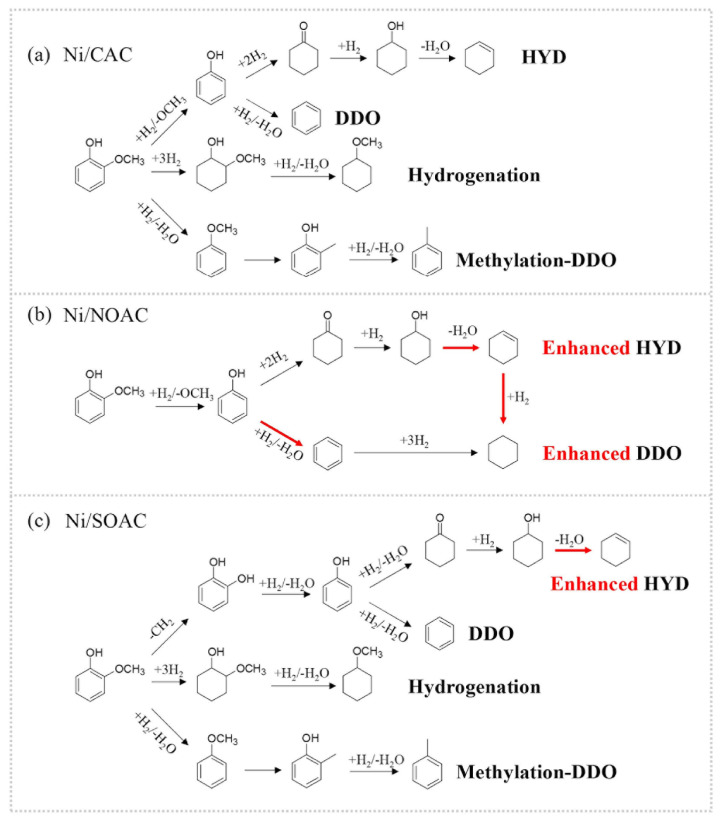
Proposed reaction routes of guaiacol on Ni/CAC (a), Ni/NOAC (b), and Ni/SOAC (c) catalysts. Note: The thick red line represents that the reaction route is promoted by the modified surface chemistry. (For interpretation of the references to colour in this figure legend, the reader is referred to the Web version of this article.) Reprinted from Ref. [77] with permission from Elsevier.

For instance, in a study by Xu et al. [77], HDO of guaiacol to biohydrocarbons was carried out via Ni catalyst and carbon support material obtained from coconut biomass (Ni/CAC). The catalyst composition was functionalized in the study with HNO_3_ (Ni/NOAC) and H_2_SO_4_ (Ni/SOA) and used in the HDO process. It has been determined that the Ni/NOAC catalyst used during the HDO process provides better benzene selectivity and lower hydrogen consumption. Additionally, the findings were supported by the results of the analysis. Due to the oxidability difference between H_2_SO_4_ and HNO_3_, the intensity of the oxygenated groups obtained from the FT-IR analysis was also measured differently. The HDO process was applied to guaiacol using each catalyst, and the mechanism was proposed, as shown in Figure 3.

**Figure 4:**
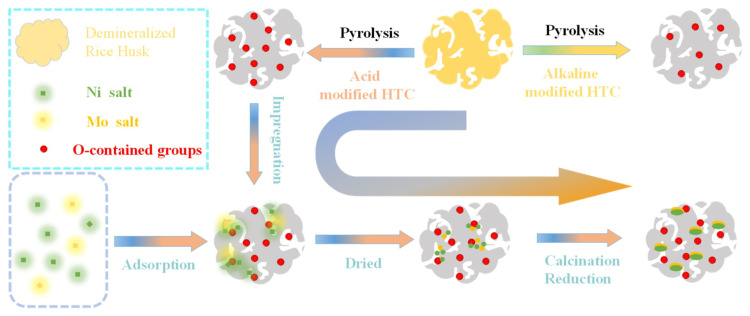
Synthesis of Ni-MoO_x_/biochar catalysts. Reprinted from Ref. [78] with permission from Elsevier.

In a study by Fan et al. [78], the HDO process was applied to bioguaiacol using Ni/MoO_x_-O_v_ interface on a rice husk char catalyst. In this work, O-containing functional groups were used to modify the surface microenvironment of Ni–Mo-loaded biomass-derived char. Rice husk was used as a biomass source and carbonized by the HTC process. Also, the surface of the rice husk biochar is enhanced with functional groups (carboxyl, carbonyl, and hydroxyl groups) that provide multiple anchorage and nucleation sites for the active metal. On the other hand, according to the product distribution of the single metal catalyst and the BTX yield, the activity of the bimetal catalysts was 25 times higher than the single metal catalysts. This result showed that new active sites were generated and considered the main factor in improving the activity of HDO. The synthesis of Ni-MoO_x_/biochar catalysts is shown in Figure 4.

**Figure 5:**
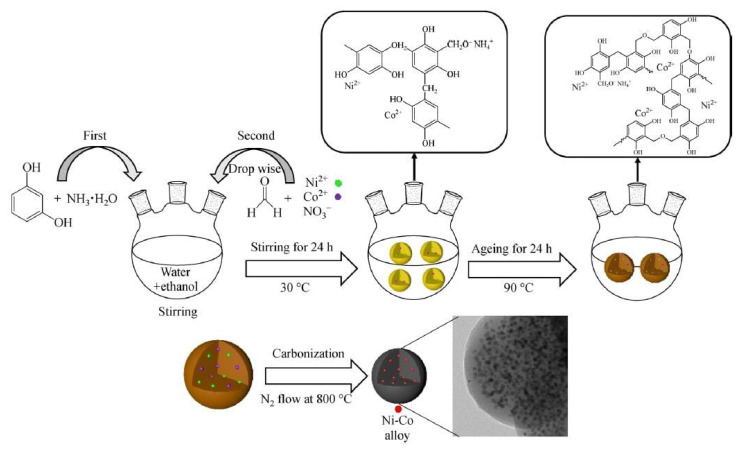
Scheme illustrating the synthesis of carbon-coated Ni-Co alloy catalysts via the extended Stöber process. Reprinted from Ref. [79] with permission from Springer.

In a study by Shi et al. [79] in 2022, HDO of methyl palmitate to hydrocarbons was carried out using methanol as a hydrogen donor. Resorcinol-formaldehyde resins were used as carbon source and Co and Ni were used as metal catalysts. Carbon-coated Ni-Co alloy catalysts were synthesized via the extended Stöber process, as shown in Figure 5. It is considered that the electrical interactions between Ni and Co and the tiny carbon microspheres are responsible for the remarkable performance of Ni-Co@C. Moreover, the metal particles demonstrated strong resistance to sintering under challenging hydrothermal conditions because of the confinement effect of carbon.

In a study conducted by Tran et al. [70] in 2020, bimetallic Mo-W carbides supported on biochar were synthesized. It was then used in the catalytic hydrotreating of canola oil at 250 °C to produce diesel grade hydrocarbons. The effects of carburization temperature and metal content on the nature of active sites were investigated using X-ray diffraction, N_2_ physisorption, X-ray photoelectron spectroscopy, and CO and H_2_ chemisorption. It was observed that the temperatures varying in the range of 550–700 °C did not have any effect on the formation of the Mo_2_C phase in bimetallic carbide. It was concluded that the addition of W to the molybdenum carbide system strongly improves catalytic performance with >95% conversion and >76% hydrocarbon yield on all mixed metal carbides at 250 °C mild temperature. On the other hand, mixed metal carbides had a higher ratio of H-regions to CO-regions than monometallic carbides.

In a study conducted by Hita et al. [80] in 2020, olive stone was used as a biomass source. Also, a multitechnical methodology is also proposed to evaluate the full spectrum of HDO reactants and products. Then, the effect of different catalysts on HDO of a crude biooil obtained from black poplar was then investigated. Three activated carbon supported catalysts based on PtPd, NiW and CoMo mixed with a commercial HZSM-5 zeolite was used. The evolution of heavy oxygenates and aromatics present in the organic liquid product fraction was monitored to gather valuable structural information about the parent compounds found in these fractions, which are also the most resistant and heavy to convert. It has been inferred that evaluating the cumulative conversions and carbon distribution of oxygenates provides a more reliable and systematic way to compare catalyst activity. As a result, phenol was obtained with 100% conversion and 45.8% selectivity.

In a study by Tran et al. [81], Ni/γ - Al_2_O_3_ and Fe/activated carbon catalysts were prepared by the initial impregnation method and then used for HDO treatment of guaiacol. Using 10% by weight of Fe / activated carbon catalyst at 300 °C and atmospheric pressure, 91.52% of guaiacol in liquid phase was successfully converted to cresol and 1,2-dimethoxybenzene products. Also, under the same reaction conditions, 96.88% guaiacol conversion was obtained using 10% by weight Ni/γ - Al_2_O_3_ catalyst. In addition, 13.03% cresol, 58.98% 1,2-dimethoxybenzene and 27.99% 3-methyl guaiacol products were obtained. The proposed reaction pathways are based on analysis of product distributions but showed that only deoxylation and transalkylation reactions occur when Fe catalysts are used. In contrast, Ni catalysts induced deoxygenation of guaiacol by deoxygenation and transalkylation reactions as well as dehydration.

## 11. Conclusion and future perspective

In this study, the effect of green carbon-based catalyst surface microenvironment on the HDO treatment applied to biooil and the reflections on its conversion to high-value chemicals were examined in detail. In particular, knowledge about current studies carried out recently has been collected, discussed in depth, and interpreted. When the current literature is examined, notably activated carbons obtained from waste-based biomass prepared with greener techniques are preferred instead of commercial and expensive carbon materials such as graphite, carbon nanofibers, carbon nanotubes, fullerene, graphene, and its derivatives. It is predicted that these carbon materials’ high dispersion encourages the formation of small particle sizes and the distribution of active metal phases, increasing exposure to active sites and enhancing the catalytic activity, because these carbon materials have a high surface area and mesoporous structure distribution. Also, the structure of the carbon support materials’ pores and their pore size distribution seriously influence the transfer of products and the adsorption of reactive agents, which directly impacts the reaction selectivity and catalytic activity. The placement of atoms such as P, O, and N on the catalyst surface with different doping methods can improve the alkaline properties, acidity, and hydrophilicity of the carbon catalyst, increasing the dispersion of active metal phases and the interaction between the catalyst surface and reactive agents in polar solvents such as water. Surface functional groups and carbon material conductivity directly influence electron transfer over reaction catalysts. Although the design of high catalytic performance carbon-based catalysts is appealing, there are several practical challenges. Green carbon-based catalysts support the rapid development of efficient and effective use of biomass resources and are promising in terms of clean fuel production. Although the preparation and application of these environmentally friendly catalysts are still limited to the laboratory stage, it is imperative to develop studies to overcome these challenges. The large scale production and use of biomass-based carbon catalysts are still considered a significant problem.

## Figures and Tables

**Table:** Latest applications of HDO for clean fuel.

Ref.	Publ. year	Catalyst	Carbon source	Carbonization technique	HDO reactant	Pressurized/continuous	H_2_ pressure (bar)	HDO temperature (ºC)	Selectivity	Conversion	Catalyst life	Side products
[82]	2022	MoO_2_ deposited porous carbon/Pd deposited porous carbon	Lignin	Nanocasting method using a silica (SiO_2_) template.	Lignin-derived guaiacol	Pressurized	30	320	85.37% phenol	<25%	54.75%	Benzene, anisole, cyclohexanol, cyclohexanone
[83]	2021	NiMo carbide supported on algal-derived activated carbon	Alg	Hydrothermal liquefaction	Algal crude biooil	Pressurized	30	350–450	73.7% phenol	98.40%	-	Cyclohexanone, cyclohexanol
[84]	2020	Nitrogen-doped carbon-supported palladium	Glucose	Hydrothermal	Guaiacol	Continuous	-	300–500	64.9% alkanes	94% Oxygen Removal	-	Short aliphatics
[85]	2021	C o –CeO_2_@NC	Chitosan	Pyrolysis	Vanillin	Pressurized	10	170	85.1% benzene	100%	6-h high benzene selectivity	Phenol
[78]	2022	Ni/Mo-doped biochar	Rice-husk	HTC + pyrolysis	Guaiacol	Continuous	-	350	99% > 2-methoxy-4-methylphenol	> 99%	5 runs 100%	-
[86]	2023	Ru-based biochar + HSiW	Forest sawdust	Pyrolysis	Guaiacol	Continuous	-	120 – 200	99.9% Cyclohexane	100%	3 runs > 87%	Cyclohexane, cyclohexanol, methoxy-cyclohexane, methoxy-cyclohexanol
[87]	2020	CoMo/ACP	Olive stone	Pyrolysis	Sawdust oil	Pressurized	65	425–475	-	-	-	-
[88]	2022	Cobalt and iron on activated carbon	Bamboo	Pyrolysis + activation	Sawdust oil	Pressurized	25–60	300–350	20.40% Phenol	-	-	Guaiacol
[89]	2021	Pd -doped biocarbon	Biomass tar	Pyrolysis + activation	Vanillin	Pressurized	H_2_ Donor (PMHS)	45–65	94.6% p-creosol	95.00%	4 runs 80.6%	High selectivity
[90]	2021	Ni-V modified Biochar	Pine nut shell, rice husk, and eucalyptus sawdust, chlorella	Hydrothermal + pyrolysis	Guaiacol	Continuous	-	380	69.17% aromatic	44.90%	3 runs 58.77% Selectivity	Phenol, anisole, cyclohexanone, cyclohexanol
[91]	2022	Pd/biochar	Glucose, corncob, pine wood chip, xylose, sucrose, and cellulose	Solvent-free carbonization + pyrolysis	Vanillin	Pressurized	20	90	53.7% levulinic acid	95.30%	After 3 h decrease	1-hydroxy-2-hexanone, 2-methyl-cyclopentanone, ethanol, sorbitol
[92]	2023	Ni/biochar catalyst	Wood	Pyrolysis + activation	Vanillin	Pressurized	30–50	100–150	> 45.6% alkanes	> 99.9% cellulose	After 24 h decrease	Sorbitol, glucose, glycerol, 1,2 -propanediol, ethylene glycol, n-propanol, isopropanol, ethanol, methanol
[93]	2020	Co_8_Ni_2_/NC	Chitin	Precarbonization + pyrolysis	Vanillin	Pressurized	5–20	30–150	90% vanillyl alcoholz	100%	Decreased selectivity in the second experiment	High selectivity
[94]	2020	N-doped carbon-supported Fe	Glucose	Pyrolysis	M-cresol	Nonpressurized	Just purged	350	-	-	-	-
[95]	2022	Mo_2_C catalysts were promoted with Ni or Pd and supported on activated biochar	Spruce, pine, and fir pellets	Pyrolysis + thermochemical activation + carbothermal reduction	Dibenzofuran	Continuous	41	230–350	90% dibenzofuran	100%	>80% coke	Phenol, esters, hydrocarbons, ketones, alcohols
[96]	2020	AC-supported Ni	Raw rice husks	Hydrothermal carbonization (HI), pyrolysis-acidification (PAI), pyrolysis-steam activation (PSI), pyrolysis (PI), pyrolysis impregnation- steam activation (PIS) and acidification-pyrolysis (API)	Guaiacol	Continuous	-	350	11.4% hydrocarbon	100%	-	Phenol, benzene, cyclohexanone, cyclohexanol, benzene, 1 – 2–dimethoxy
[70]	2020	Mo-W carbides supported on biochar	Spruce, fir, and pine	Vacuum pyrolysis	Vegetable oil (canola oil	Continuous	-	250	87% hydrocarbon	100%	2 runs 60% conversion and 78% selectivity	N- octadecane, n-heptadecane
[97]	2020	Carbon-covered alumina-supported Ni_2_P	Sucrose	Incipient wetness impregnation + calcination	Pyrolysis oil	Pressurized	50–100	150–250	< 20% hydrocarbon	59.50%	-	Phenols, ketones, acids
[98]	2019	Mo_2_C/activated biochar	Spruce, pine, and fir pellets	Pyrolysis + activation	4-methylphenol	Pressurized	43	350	97% methylcyclohexane	84%	-	-
[80]	2020	ACP (phosphor containing activated carbon) supported PtPd, NiW, and CoMo	Olive stone	Chemical activation	Black poplar	Continuous	65	450	45.8% phenolics	100%	-	Ketones, phenolics, acids, esters, hydrocarbons, acetic acid, methanol, acetone
[99]	2020	Ru/C	Starch/glucose/saw dust and alpha cellulose	Single-step calcination in nitrogen	Microalgae oil	Pressurized	50	140	88% heptadecane, 12% octadecane	100%	Stable at least 8 runs	1-octadecanol, esters
[100]	2019	NiCu/biochar	Cellulose	Ionic liquid treatment + pyrolysis	5-hydroxymethylfurfural	Pressurized	10–40	100–220	63.8% 5-methylfurfural	62.70%	Stable at least 5 runs	2,5-dihydroxymethylfuran, dimethylfurfural
[101]	2020	CoAlRu-LDH/C	Glucose	Hydrothermal	Guaiacol	Pressurized	5–20	180–260	97.3% cyclohexanol	97.80%	Stable at least 5 runs	Benzene, cyclohexanone, cyclohexane, cyclohexanediol
[81]	2021	Fe/AC and Ni/γ-Al_2_O_3_	Bamboo	Carbonization and activation	Guaiacol	Continuous	-	300	95.93% 1,2-dimethoxy benzene	87.09%	-	3-methly guaiacol, o-cresol
[82]	2022	MoO_2_ deposited porous carbon/Pd deposited porous carbon	Lignin	Nanocasting method using a silica (SiO_2_) template.	Lignin-derived guaiacol	Pressurized	30	320	85.37% phenol	<25%	54.75%	Benzene, anisole, cyclohexanol, cyclohexanone
[83]	2021	NiMo carbide supported on algal-derived activated carbon	Alg	Hydrothermal liquefaction	Algal crude biooil	Pressurized	30	350–450	73.7% phenol	98.40%	-	Cyclohexanone, cyclohexanol
[84]	2020	Nitrogen-doped carbon-supported palladium	Glucose	Hydrothermal	Guaiacol	Continuous	-	300–500	64.9% alkanes	94% Oxygen Removal	-	Short aliphatics
[85]	2021	C o –CeO_2_@NC	Chitosan	Pyrolysis	Vanillin	Pressurized	10	170	85.1% benzene	100%	6-h high benzene selectivity	Phenol
[78]	2022	Ni/Mo-doped biochar	Rice-husk	HTC + pyrolysis	Guaiacol	Continuous	-	350	99% > 2-methoxy-4-methylphenol	> 99%	5 runs 100%	-
[86]	2023	Ru-based biochar + HSiW	Forest sawdust	Pyrolysis	Guaiacol	Continuous	-	120 – 200	99.9% Cyclohexane	100%	3 runs > 87%	Cyclohexane, cyclohexanol, methoxy-cyclohexane, methoxy-cyclohexanol
[87]	2020	CoMo/ACP	Olive stone	Pyrolysis	Sawdust oil	Pressurized	65	425–475	-	-	-	-
[88]	2022	Cobalt and iron on activated carbon	Bamboo	Pyrolysis + activation	Sawdust oil	Pressurized	25–60	300–350	20.40% Phenol	-	-	Guaiacol
[89]	2021	Pd -doped biocarbon	Biomass tar	Pyrolysis + activation	Vanillin	Pressurized	H_2_ Donor (PMHS)	45–65	94.6% p-creosol	95.00%	4 runs 80.6%	High selectivity
[90]	2021	Ni-V modified Biochar	Pine nut shell, rice husk, and eucalyptus sawdust, chlorella	Hydrothermal + pyrolysis	Guaiacol	Continuous	-	380	69.17% aromatic	44.90%	3 runs 58.77% Selectivity	Phenol, anisole, cyclohexanone, cyclohexanol
[91]	2022	Pd/biochar	Glucose, corncob, pine wood chip, xylose, sucrose, and cellulose	Solvent-free carbonization + pyrolysis	Vanillin	Pressurized	20	90	53.7% levulinic acid	95.30%	After 3 h decrease	1- hydroxy-2-hexanone, 2-methyl-cyclopentanone, ethanol, sorbitol
[92]	2023	Ni/biochar catalyst	Wood	Pyrolysis + activation	Vanillin	Pressurized	30–50	100–150	> 45.6% alkanes	> 99.9% cellulose	After 24 h decrease	Sorbitol, glucose, glycerol, 1,2 -propanediol, ethylene glycol, n-propanol, isopropanol, ethanol, methanol
[93]	2020	Co_8_Ni_2_/NC	Chitin	Precarbonization + pyrolysis	Vanillin	Pressurized	5–20	30–150	90% vanillyl alcohol	100%	Decreased selectivity in the second experiment	High selectivity
[94]	2020	N-doped carbon-supported Fe	Glucose	Pyrolysis	M-cresol	Nonpressurized	Just purged	350	-	-	-	-
[95]	2022	Mo_2_C catalysts were promoted with Ni or Pd and supported on activated biochar	Spruce, pine, and fir pellets	Pyrolysis + thermochemical activation + carbothermal reduction	Dibenzofuran	Continuous	41	230–350	90% dibenzofuran	100%	>80% coke	Phenol, esters, hydrocarbons, ketones, alcohols
[96]	2020	AC-supported Ni	Raw rice husks	Hydrothermal carbonization (HI), pyrolysis-acidification (PAI), pyrolysis-steam activation (PSI), pyrolysis (PI), pyrolysis impregnation- steam activation (PIS) and acidification-pyrolysis (API)	Guaiacol	Continuous	-	350	11.4% hydrocarbon	100%	-	Phenol, benzene, cyclohexanone, cyclohexanol, benzene, 1 – 2–dimethoxy
[70]	2020	Mo-W carbides supported on biochar	Spruce, fir, and pine	Vacuum pyrolysis	Vegetable oil (canola oil	Continuous	-	250	87% hydrocarbon	100%	2 runs 60% conversion and 78% selectivity	N- octadecane, n-heptadecane
[97]	2020	Carbon-covered alumina-supported Ni_2_P	Sucrose	Incipient wetness impregnation + calcination	Pyrolysis oil	Pressurized	50–100	150–250	< 20% hydrocarbon	59.50%	-	Phenols, ketones, acids
[98]	2019	Mo_2_C/activated biochar	Spruce, pine, and fir pellets	Pyrolysis + activation	4-methylphenol	Pressurized	43	350	97% methylcyclohexane	84%	-	-
[80]	2020	ACP (phosphor containing activated carbon) supported PtPd, NiW, and CoMo	Olive stone	Chemical activation	Black poplar	Continuous	65	450	45.8% phenolics	100%	-	Ketones, phenolics, acids, esters, hydrocarbons, acetic acid, methanol, acetone
[99]	2020	Ru/C	Starch/glucose/saw dust and alpha cellulose	Single-step calcination in nitrogen	Microalgae oil	Pressurized	50	140	88% heptadecane, 12% octadecane	100%	Stable at least 8 runs	1-octadecanol, esters
[100]	2019	NiCu/biochar	Cellulose	Ionic liquid treatment + pyrolysis	5-hydroxymethylfurfural	Pressurized	10–40	100–220	63.8% 5-methylfurfural	62.70%	Stable at least 5 runs	2,5-dihydroxymethylfuran, dimethylfurfural
[101]	2020	CoAlRu-LDH/C	Glucose	Hydrothermal	Guaiacol	Pressurized	5–20	180–260	97.3% cyclohexanol	97.80%	Stable at least 5 runs	Benzene, cyclohexanone, cyclohexane, cyclohexanediol
[81]	2021	Fe/AC and Ni/γ-Al_2_O_3_	Bamboo	Carbonization and activation	Guaiacol	Continuous	-	300	95.93% 1,2-dimethoxy benzene	87.09%	-	3-methly guaiacol, o-cresol
